# Anthropometric, Lifestyle Characteristics, Adherence to the Mediterranean Diet, and COVID-19 Have a High Impact on the Greek Adolescents’ Health-Related Quality of Life

**DOI:** 10.3390/foods11182726

**Published:** 2022-09-06

**Authors:** Stamatina Papadaki, Vilelmine Carayanni, Venetia Notara, Dimitrios Chaniotis

**Affiliations:** 1 Department of Public and Community Health, University of West Attica, 11521 Athens, Greece; 2 Department of Tourism Management, University of West Attica, 12243 Athens, Greece; 3 Department of Biomedical Sciences, University of West Attica, 12243 Athens, Greece

**Keywords:** health-related quality of life, Mediterranean diet, body mass index, lifestyle, sedentary habits, adolescents, COVID-19

## Abstract

Objective: The study aimed at analyzing the relationship between anthropometric characteristics, lifestyle, and dietary habits, as well as the burden of the pandemic on the health-related quality of life among Greek pupils. Research methods and procedures: On the whole, 2088 adolescents aged 12–18 years from Attica, Greece, were enlisted in this school-based cross-sectional study that took place in May–December 2021. Health-related quality of life was estimated through the KIDSCREEN-27 questionnaire, adherence to the Mediterranean diet—through the KIDMED test. For the empirical and econometric analyses, the Mann–Whitney U and Kruskal–Wallis means comparison tests were utilized; multiple linear regression was used accordingly. Results: The present study provides evidence to the fact that boys, younger adolescents, adolescents living with both parents and with highly educated mothers had a better health-related quality of life. Concerning their eating practices, positive predictors were consuming a better-quality breakfast, having all five meals daily, consuming lunch and dinner with parents, and higher adherence to the Mediterranean diet. Moreover, sufficient night sleep time, fewer hours spent on screen viewing, more frequent walks, and having hobbies were linked to the health-related quality of life with a positive sign. In contrast, negative predictors were higher body mass index and everyday life difficulties due to the COVID-19 pandemic crisis. Conclusions: Greek adolescents’ anthropometric characteristics, BMI, lifestyle and sedentary habits, eating habits, and adherence to the Mediterranean diet were significantly related to their perceived health-related quality of life during the pandemic.

## 1. Introduction

In recent years, COVID-19 has impacted the mental balance of vulnerable population groups [1], both children’s and adolescents’ behavior as well as psychological stability [2] and caused great concern and stress in their daily lives [3]. Studies have reported feelings of loneliness and depression [3], increased anxiety [4], high risk of mental health disorders, and an overall lower quality of life [5]. Adolescents’ health and well-being are important determinants for the later life health and the growth and development of societies. Therefore, attention should be paid during all stages of the lifecycle [6]. Protection of the adolescents’ health has triple public health benefits: for the adolescents’ current life, for their adult life, and for the future life through their offspring [7]. Health status is linked with many aspects of general welfare and quality of life [8]; thus, improving the health-related quality of life (HRQoL) among youths should be a health priority.

With a view to measure adolescents’ perceived health and general well-being, HRQoL has been used in numerous studies [9,10,11]. In particular, HRQoL presents an overview of physical, psychological, and social welfare [8,12] and has been widely used to investigate children’s and adolescents’ mental health disorders [13]. Nutrition together with diet are major health determinants, while COVID-19 has had an intense impact on the adolescents’ dietary habits [14]. An unhealthy dietary pattern can lead to future negative health outcomes like overweight or obesity [15], cardiovascular diseases, diabetes, [16] and lower HRQoL [17]. On the other hand, adhering to a quality diet and adopting healthy eating habits provides a series of health benefits. In particular, Mediterranean diet (MD) is related to better weight management against childhood overweight/obesity [18] and positively associated with higher academic performance [19,20] and overall better HRQoL [21]. Consuming breakfast is also linked to higher overall dietary quality and better food choices [22]. Breakfast is considered to be of major importance; an ideal breakfast should contain low-fat milk/milk-derived products, unrefined grains, and fresh fruit/fruit juice [23]. Systematic breakfast consumption has a protective effect against obesity [24], is positively associated with school performance [25] and better quality of life [26]. In addition, Ferrer-Cascales et al. [27] revealed that adolescents who consumed more nutritious breakfasts had a better HRQoL and less stress and feeling of depression.

Furthermore, insufficient physical activity is a health risk. Over the past two years, during the pandemic crisis, studies have confirmed increased sedentary behavior. This involves screen viewing, watching television, using smartphones, tablets, and computers for reading, communication with the use of popular social platforms, playing electronic games [3,28], and, in the meantime, less time spent on physical activities (PA) [29,30]. This unhealthy lifestyle, with less PA and more sedentary habits, is associated with a lower HRQoL [26,31]. Lower HRQoL among youths during the pandemic has also been confirmed in recent studies [32,33].

To the best of the authors’ knowledge, information and research related to the burden of COVID-19 on the Greek adolescents’ everyday life and the associations between diet, sedentary lifestyle, several other lifestyle factors such as sleep duration, having hobbies and HRQoL is limited. Thus, the main aim of this study was to fill this gap and enrich the empirical literature. In particular, the current study investigates and tests two hypotheses. The initial hypothesis was based on the assumption that higher adherence to the Mediterranean diet (AMD), healthier eating habits and lifestyle characteristics are positively related to HRQoL. The second hypothesis considered that difficulties arising in everyday life due to the COVID-19 crisis worsen the adolescents’ HRQoL. To validate and test these two hypotheses, a sample of adolescents residing in a Mediterranean country, Greece, in the Attica region, was investigated. Empirical findings validated the mentioned hypotheses.

## 2. Materials and Methods

### 2.1. Study Design

In all, 2088 adolescents aged 12–18 years old from the Attica region of Athens took part in this cross-sectional school-based research that was contacted from May to December 2021. The proportionate random stratified sampling method was applied based on the secondary students’ population (data from school year 2018/2019) according to the Hellenic Statistical Authority. Initially 2700 questionnaires were shared between several public high schools from all the seven mainland regions of Attica, that is, from Central Athens, South Athens, North Athens, West Athens, West Attica, East Attica, and Piraeus, proportionally to the overall number of schoolchildren. To achieve a high level of sample representation, the sample size calculation was in accordance with a 5% margin of error, a 95%, confidence level, 109,400 total student population, and a 50% response distribution. The acceptance rate was around 77%. The adolescents were briefed about the purpose of the research and participated voluntarily during classes. Written informed consent forms were signed by the parents/guardians and collected prior to the distribution of the questionnaires. The main researcher was present to offer guidance and instructions about the questionnaire. The questionnaire duration was around 30 min. The Institute of Educational Policy of Greece (59235/2021) and the West Attica University Research Ethics Committee (18092-03/03/2021) approved this study.

### 2.2. Instruments

A structured questionnaire was created and administered to all the adolescents with general questions involving anthropometric data, family characteristics, eating habits, lifestyle and sleep patterns. Furthermore, questions about the burden of COVID-19 on their everyday life or the life of the family were included. A pilot test was conducted among 50 pupils, who were excluded from the final analysis, to detect any unclear questions.

In particular, the anthropometric questions were about sex, age, weight, and height, and the self-reported anthropometric data were evaluated to calculate the BMI (kg/m^2^). After that, the adolescents were classified accordingly as underweight, normal weight, overweight, and obese based on the sex- and age-specific BMI cutoff points of the International Obesity Task Force (IOTF) [34,35].

Moreover, the adolescents were questioned about their nationality, family structure (“live with both parents”, “live with one parent”, “live with grandparents”, “other”), and parental educational and employment status. Furthermore, questions were asked about the frequency of going for a walk and the frequency of getting involved with hobbies (“never”, “once every month”, “1–2 times every week”, “3–5 times every week”, “almost every day”). Information about their daily average hours of night sleep was acquired. The adolescents also reported the hours they spent on screen viewing during the week and on weekends (“none at all”, “about 1 h”, “about 2–3 h”, “about 4 h”, “more than 4 h”).

Regarding eating habits, the questions about the choices over breakfast had the following possible responses: “I consume ‘only a glass of milk/dairy product’/‘a glass of milk/dairy product and grains/cereals and fruit/fruit juice’/‘sweets like donuts/cakes/biscuits’/‘puff pastry/pies, sandwiches’”. The questions also involved information about the number of meals the adolescents consumed every day and the number of meals consumed together with their parents.

In order to identify any consequences COVID-19 has had on them and/or their families, the adolescents were asked the following yes–no questions: “During the pandemic period, ‘has your mother lost her job’/‘has your father lost his job’/‘have you been short of money to pay rent, bills, heating’/‘have you been short of money to buy food’/‘has COVID-19 caused quarrels within the family’/‘has COVID-19 created psychological distress such as anxiety, panic, growing anxiety, uncertainty about the future’/‘has COVID-19 created difficulties in education including inability to pursue distance learning, interruption of private tuition’”.

HRQoL was evaluated using the KIDSCREEN-27 questionnaire [36]. KIDSCREEN-27 is a reliable and widely applied tool validated for the Greek population [37]. The Greek-language self-report version for adolescents was used. The instrument measures five dimensions: physical well-being (five questions), psychological well-being (seven questions), parent relations and autonomy (seven questions), social support and peers (four questions), and school environment (four questions). Each of the questions is scored on a five-point scale (“not at all”, “a little”, “moderately”, “much”, or “very much”). Higher scores are linked to better HRQoL.

Finally, the AMD was assessed using the KIDMED test [38], which was translated into the Greek language using the forward–backward translation method. This widely applied tool consists of 16 yes–no questions. Four of the questions with negative indication to the MD are assigned –1 and twelve questions with positive indication are assigned +1. The KIDMED test classifies individuals (KIDMED index) into three categories. The diet quality is low if the score is < 3, medium adherence to the MD is confirmed if the score is between 4 and 7, and optimal adherence is confirmed if the score is > 8.

### 2.3. Data Analysis

Analysis was performed using the STATA 16 software. For descriptive statistics, we used frequencies (N), percentages, means, and standard deviations. Cronbach’s alpha coefficient was used to evaluate the KIDSCREEN-27 questionnaire’s internal consistency. The Mann–Whitney U and Kruskal–Wallis means comparison tests were applied to two or more clusters, respectively. For the econometric analysis, the multiple linear regression approach was employed to analyze the impact of anthropometric and lifestyle characteristics and the AMD on the adolescents’ HRQoL. In particular, the following equation was assessed:

$$HRQoL_{i}=a_{i}+bX_{i}+e_{i}$$

where $HRQoL_{i}$ is the KIDSCREEN-27’s total score of each adolescent, $a_{i}$ is the constant term, $X_{i}$ is the vector of anthropometric and lifestyle characteristics and adherence to the Mediterranean diet, b is the estimated coefficient for every independent variable $X_{i}$ and $e_{i}$ is the error term of the estimated regression.

## 3. Results

### 3.1. KIDSCREEN-27’s Cronbach’s Alpha Tests

Cronbach’s alpha for KIDSCREEN-27 indicated acceptable reliability with a >0.7 in every dimension. The mean test scale was at a = 0.79: a was estimated to be 0.76 for the first dimension (physical health), 0.71 for the second dimension (psychological well-being), 0.72 for the third dimension (parent relations and autonomy), 0.79 for the fourth dimension (social support and peers), and 0.76 for the fifth dimension (school environment). Furthermore, having confirming that the variables were not normally distributed (Shapiro–Wilk W and Shapiro–Francia tests), we used the Mann–Whitney U and Kruskal–Wallis comparison tests for two and more clusters, respectively.

### 3.2. Sample Characteristics

The table below (Table 1) shows the adolescents’ anthropometric and socioeconomic characteristics and the AMD in total and by sex. Furthermore, the Mann–Whitney U test results are provided, highlighting the differences by sex.

In all, 50.7% were girls and 49.3% boys. The participants were between 12 and 18 years old, with the mean age of 15.1 years. The mean weight was 60.8 kg, mean height—1.69 m, mean BMI—21.2. The boys were found to significantly differ from the girls in height, BMI, and some lifestyle characteristics. More specifically, as expected, the boys weighed more (*p* < 0.001), were taller (*p* < 0.001), and had a higher BMI (*p* < 0.001) than the girls. The boys also slept more hours during the night (*p* = 0.070). Of the total sample, almost 82% were from two-parent families, around one out of two mothers had a higher educational level whereas the fathers had a 10% lower level of tertiary education. Regarding the weight categories, more boys were overweight (20.9%) and obese (5.1%) than girls (10.9% and 3.25%, respectively). On the other hand, more girls were underweight (9.6%) or had normal weight (76.2%) than boys (5.1% and 68.9%, respectively). As for the AMD, only 9.1% of the total sample had optimal adherence to the MD. The boys presented a higher percentage of medium (59.8%) and optimal (12%) adherence, whereas the girls presented a higher percentage of low (37.6%) AMD.

The following table (Table 2) presents the summary statistics for KIDSCREEN-27 by sex and eating habits.

**Table 2:** Summary statistics for the KIDSCREEN-27’s total score and all the dimensions.

Variables		KIDSCREEN-27’s Total Score	Physical Health	Psychological Well-Being	Parent Relations and Autonomy	Social Support and Peers	School Environment
Panel A: by sex							
Boys	Mean (SD)	99.486 (16.47)	48.265 (10.34)	45.838 (10.21)	46.199 (9.65)	48.514 (11.84)	44.262 (9.06)
Girls	Mean (SD)	90.208 (16.71)	41.32 (8.49)	39.752 (10.09)	43.148 (8.25)	47.338 (11.50)	43.42 (8.16)
*p*-value		<0.001	<0.001	<0.001	<0.001	0.006	0.004
Panel B: by eating habits (total sample)							
High-quality breakfast	Mean (SD)	97.37 (16.05)	46.23 (9.88)	43.84 (10.28)	45.58 (8.34)	48.58 (10.78)	44.83 (8.01)
Low-quality breakfast	Mean (SD)	94.09 (17.12)	44.08 (10.01)	42.69 (10.33)	44.46 (9.03)	47.63 (12.18)	43.64 (8.79)
*p*-value		<0.001	<0.001	0.005	0.010	0.147	0.003
Five meals/day	Mean (SD)	101.22 (16.76)	48.11 (10.89)	46.46 (11.25)	47.40 (9.88)	49.36 (11.58)	46.87 (8.38)
Fewer meals/ day	Mean (SD)	93.61 (17.06)	44.11 (9.75)	42.05 (10.35)	44.18 (8.88)	47.65 (11.68)	43.33 (8.57)
*p*-value		<0.001	<0.001	<0.001	<0.001	0.060	<0.001
Lunch with parents	Mean (SD)	99.41 (15.90)	46.29 (10.19)	45.01 (10.16)	46.96 (8.93)	49.16 (11.55)	45.59 (8.29)
Lunch alone	Mean (SD)	91.40 (17.39)	43.56 (9.83)	41.07 (10.59)	42.92 (8.79)	46.97 (11.69)	42.61 (8.63)
*p*-value		<0.001	<0.001	<0.001	<0.001	<0.001	<0.001
Dinner with parents	Mean (SD)	100.77 (15.82)	46.86 (9.83)	45.86 (10.27)	47.25 (9.19)	50.04 (11.55)	45.94 (8.64)
Dinner alone	Mean (SD)	91.20 (17.09)	43.47 (10.02)	40.86 (10.37)	43.07 (8.65)	46.61 (11.58)	42.62 (8.38)
*p*-value		<0.001	<0.001	<0.001	<0.001	<0.001	<0.001

Notes: Authors’ calculations; *p*-value refers to the Mann–Whitney U tests. Abbreviations: SD: standard deviation.

Table 2 shows the Mann–Whitney U test results for the total score and for each of the five KIDSCREEN-27 dimensions. Significant differences between the sexes were found, with the boys having a better HRQoL. Particularly, the boys had a significantly higher score in physical health (*p* < 0.001), psychological well-being (*p* < 0.001), parent relations and autonomy (*p* < 0.001), social support and peers (*p* = 0.006), school environment (*p* = 0.004), and in the KIDSCREEN-27’s total score (*p* < 0.001).

Moreover, significant differences between eating habits and HRQoL were found. In our study, the adolescents who had healthier eating habits also had a better HRQoL. Specifically, the adolescents who consumed better-quality breakfasts, had all five meals every day (breakfast, brunch, lunch, afternoon snack, and dinner), and consumed their lunch and dinner with their parents had a better HRQoL and scored significantly higher in the KIDSCREEN-27’s total score and in almost all the dimensions (*p* < 0.001).

Aiming to evaluate the consequences of COVID-19, the adolescents were asked a set of questions about the changes the pandemic caused for them or their families. Table 3 shows the statistically significant differences between KIDCREEN-27 and the pandemic crisis aftermath.

The adolescents reported that the COVID-19 crisis had negatively affected them and/or their families in various ways and that they had a significantly lower HRQoL. More specifically, the adolescents whose parents (mother/father) were left unemployed due to the pandemic crisis (almost all *p* < 0.001), those who reported that the pandemic crisis caused financial issues (almost all *p* < 0.001), food deficiency (almost all *p* < 0.001), quarrels within the family (all *p* < 0.001), worse psychology (all *p* < 0.001), and difficulties with classes (all *p* < 0.001) had a significantly lower HRQoL.

### 3.3. Ordinary Least Squares Analysis

The ordinary least squares technique was performed to investigate the association between the independent anthropometric, sociodemographic, and lifestyle variables and the HRQoL. In particular, Table 4 shows the empirical findings in reference to the multiple lineal regression models.

The above table reveals the empirical associations between the KIDSCREEN-27’s total score and several anthropometric, lifestyle, sedentary characteristics and the AMD. It was found that sex was a statistically significant parameter of the health-related quality of life and, specifically, the coefficient of sex was found to be highly significant (*p* < 0.001) in all the estimations. That result suggests that boys perceive significantly better their health-related quality of life. As far as age was concerned, it was found that the younger adolescents had a better HRQoL than the older ones. Furthermore, an adolescent’s mother’s educational status was positively related to their HRQoL, while, on the contrary, paternal educational level was found to be an insignificant factor. The BMI was negatively and statistically significant (*p* = 0.036) as correlated to the HRQoL. More specifically, it was observed that as the BMI increases by one unit, the KIDSCREEN-27’s total score decreases by 0.22 units, indicating its high negative impact on the adolescents’ life quality. Regarding their eating habits, we showed that the adolescents that had a higher AMD had a significantly higher KIDSCREEN-27’s total score (*p* < 0.001) and, therefore, a better HRQoL. Our findings support that there are many health benefits for youths who adherence to this particular diet.

Furthermore, it was estimated that healthy lifestyle habits were highly associated with HRQoL. Living with both parents had a positive impact on an adolescent’s HRQoL (*p* < 0.001), whereas more time spent on screen viewing (use of television, phone, and other electronical devices) resulted in a significantly lower HRQoL. With regard to night sleep duration, the majority of the estimated models showed that a nonlinear relationship exists between night sleep hours and perceived HRQoL following an inverse U-shaped relationship. More specifically, it was found that the adolescents who slept more at night had a significantly higher KIDSCREEN-27’s total score, but after a threshold point (around 11.4 h), this relationship turned around. Finally, the empirical results showed that a more social lifestyle was related to better HRQoL. In particular, the students who went out for a walk frequently and had hobbies had a better health-related quality of life. The R-squared statistic is equal to 0.26, indicating that around 26% of the HRQoL variance is explained by the set of the independent variables used in the analysis.

## 4. Discussion

This cross-sectional study provided evidence that anthropometric, lifestyle, and sedentary characteristics as well as the AMD and other eating habits are important determinants of the HRQoL among Greek adolescents during the COVID-19 pandemic. A sample of 2088 adolescents in the Attica region was investigated while the empirical analysis was based on descriptive measures, nonparametric approaches, and regression techniques.

Sex and age were found to be positive predictors of the HRQoL. In this study, similarly to previous findings, the boys [21,39,40,41,42,43,44] and the younger adolescents [39,42,44,45,46] had a better HRQoL. We expected these findings as sex and age differences in the health-related quality of life are confirmed and could be connected to puberty with all the biological, emotional, and social changes that occur during this period.

In addition, family structure has a key role in adolescents’ well-being and health. Torsheim et al. [47] showed that adolescents not living with their parents perceive their health worse, while Barbieri et al. [46] showed that single parenthood could increase mental health issues. In this study, it was revealed that the adolescents who lived with both their parents had a better HRQoL, a finding reported previously among youths [21,48]. Moreover, the role of the maternal educational status with respect to the HRQoL has been observed [49,50]. Similarly, in the present study, a higher education level amongst the mothers resulted in a better HRQoL of the adolescents. We could assume that better educated mothers are better informed about health-related issues and could, therefore, encourage a healthier lifestyle and/or support the adolescents’ behavioral disorders.

Healthy eating practices are positively related to the HRQoL [17,40,51]. In agreement with the previous results, the present study revealed that the adolescents who consumed a better-quality breakfast [26,27], those who consumed all five meals daily [52], had lunch and dinner with their family [49,52], and those who had optimal AMD [21,28,43,49,52,53,54] had a better HRQoL. It is known that the higher diet quality that the MD provides, along with other healthy eating habits like breakfast consumption, offers various physical health benefits. In addition, with our study, we showed that the MD and adhering to healthy eating practices are linked to a better health-related quality of life and the overall adolescents’ psychological well-being.

Furthermore, it is known that adolescents with a healthier lifestyle have a better HRQoL [53,55]. In good match with the previous studies, it was also proved that the adolescents spending less time on sedentary habits and using screens less [21,43,56,57] had a better HRQoL. According to this research, the adolescents who went out for a walk frequently and the adolescents who had hobbies also had a better HRQoL. Additionally, sleep quality and good sleep duration have been associated with a better HRQoL in adolescents [26,43]. We found that the adolescents who slept more during the night had a better HRQoL, as also reported in the previous studies [21,55,57]. However, it is of high interest that this study found that sleep duration has a nonlinear effect on the HRQoL. In particular, sleep duration up to around 11 h per night is positively associated with the HRQoL, while after this threshold, its effect turns to negative.

On the other hand, a higher BMI was a negative predictor of the HRQoL. The previous research also supported that youths with a higher BMI and excess weight have a lower HRQoL [52,53,58,59,60]. Overweight and obese youths may suffer from health difficulties or may be criticized and bullied about their body image. That could lead to dissatisfaction and feelings of disappointment, lower self-esteem, stress, depression, and an overall impaired HRQoL. It is very important for policymakers when promoting health programs to bear in mind that excess body weight impacts the overall welfare of adolescents and lowers their HRQoL.

Finally, as expected, COVID-19 was negatively associated with the HRQoL, an essential finding in line with other studies [28,32,33,46,61]. The adolescents who stated that their parents were left unemployed due to the COVID-19 crisis, their family had financial issues, experienced food insecurity, faced arguments in the family [61], had worse psychology [28,46], or difficulties with classes had a lower HRQoL. It is important to highlight that besides the pandemic’s negative physical health outcomes, COVID-19 has also impacted the mental health of youths and caused major psychological disorders and a lower quality of life.

This research has important policy implications. First, the findings suggest that healthy eating habits and healthy lifestyle recommendations for youths should be a health priority. Schools, the community, and families should reinforce positive diet behaviors like regularly consuming quality breakfast and having an optimal AMD. Furthermore, it is important for families to create safe and supportive environments to encourage youths to lead a positive lifestyle with fewer sedentary habits, less use of screens, more social life, and more hobbies. Finally, parents or guardians should be trained to offer psychosocial support and manage adolescents’ behavioral disorders.

Nonetheless, when interpreting the findings of this study, there are some potential shortcomings that should be acknowledged. Firstly, it is a cross-sectional study and does not allow us to estimate the causal relationships between the variables. Panel data are necessary to investigate the time series relationships between the outcome variables and the previously mentioned regressors. Secondly, the anthropometric data were self-reported, and research has revealed that sometimes weights and heights are misreported [62]. Thirdly, one geographical area of Greece, the region of Attica, was covered; thus, it is difficult to make country generalizations.

## 5. Conclusions

This study enhances the comprehension of the impact of adolescents’ anthropometric characteristics, lifestyle, AMD, eating habits, and COVID-19 on their HRQoL. To the best of the researchers’ knowledge, this study was the first conducted in a Mediterranean region in Attica, Greece, during the pandemic providing evidence of the importance of adhering to the MD, having fewer sedentary habits, and a healthier lifestyle for achieving a better HRQoL in adolescents. Furthermore, we found that COVID-19 may have a negative impact on the youth-perceived well-being and health-related quality of life. The scientific importance of this study is that the implications of the relationship between anthropometric characteristics and the adolescents’ healthy lifestyle and AMD are a major increasing concern for policymakers in view of their effect on the youths’ HRQoL and well-being. Future research should investigate a wider sample population. Additionally, employing longitudinal data would provide reliable estimates that will take into account long-term youth behavior.

## Figures and Tables

**Table 1:** Anthropometric and socioeconomic characteristics and the AMD by sex.

**Panel A**				
**Variables**	**Total** **Mean (SD)**	**Boys** **Mean (SD)**	**Girls** **Mean (SD)**	***p*-Value**
Age	15.07 (1.46)	15.00 (1.51)	15.14 (1.41)	0.077
Height	1.69 (0.10)	1.74 (0.09)	1.64 (0.07)	<0.001
Weight	60.78 (13.3)	65.6 (14.49)	55.97 (9.85)	<0.001
BMI	21.23 (3.68)	21.61 (3.98)	20.85 (3.30)	<0.001
Night sleep duration	7.40 (1.15)	7.44 (1.17)	7.35 (1.12)	0.070
**Panel B**				
**Variables**	**Total N (%)**	**Boys N (%)**	**Girls N (%)**	
Sex	2081 (100)	1025 (49.3)	1052 (50.7)	
**Family structure**				
Single parent	282 (13.9) *	128 (12.9)	151 (14.8)	
Two parents	1666 (81.8)	809 (81.2)	856 (82.4)	
Other	84 (4.3)	59 (5.9)	29 (2.8)	
**Maternal educational level**				
Primary	195 (9.6)	90 (9.1)	105 (10.2)	
Secondary	733 (36.2)	372 (37.7)	360 (34.8)	
Tertiary	1095 (54.2)	525 (53.2)	568 (55.0)	
**Paternal educational level**				
Primary	264 (13.2)	126 (12.8)	138 (13.7)	
Secondary	864 (41.4)	401 (40.8)	424 (42.0)	
Tertiary	905 (45.4)	456 (46.4)	447 (44.3)	
**Weight categories**				
Underweight	141 (7.38)	49 (5.11)	92 (9.65)	
Normal weight	1386 (72.53)	660 (68.89)	726 (76.18)	
Overweight	304 (15.91)	200 (20.88)	104 (10.91)	
Obese	80 (4.19)	49 (5.11)	31 (3.25)	
**Adherence to the MD**				
Low	181 (32.9)	271 (28.11)	383 (37.59)	
Medium	1150 (57.93)	577 (59.85)	571 (56.04)	
Optimal	181 (9.12)	116 (12.03)	65 (6.38)	

Notes: Authors’ calculations. Panel A: means and standard deviations (in parentheses) are presented; *p*-value refers to the Mann–Whitney U test. Panel B: Frequencies are presented. Abbreviations: BMI: body mass index. * 13% of the sample live with their mother, 0.9%—with their father.

**Table 3:** KIDSCREEN-27’s total score and the five dimensions with respect to several variables related to the COVID-19.

Due to COVID-19	KIDSCREEN-27’s Total Score	*p*-Value	Physical Health	*p*-Value	Psychological Well-Being	*p*-Value	Parent Relations and Autonomy	*p*-Value	Social Support and Peers	*p*-Value	School Environment	*p*-Value
Unemployed (mother)	95.11	<0.001	44.82	0.07	42.96	<0.001	44.89	<0.001	47.98	0.07	43.94	0.08
	85.58		42.44		36.89		38.95		45.59		41.91	
Unemployed (father)	95.21	<0.001	44.94	<0.001	43.01	<0.001	44.84	<0.001	48.07	<0.001	43.98	<0.001
	83.15		40.14		36.38		39.43		43.70		40.29	
Short of money for rent/bills/heating	95.61	<0.001	45.03	<0.001	43.18	<0.001	45.18	<0.001	48.06	0.02	44.07	<0.001
	84.00		40.86		37.26		38.21		46.02		40.87	
Short of money for food	95.16	<0.001	44.84	0.01	42.94	<0.001	44.94	<0.001	48.01	0.01	43.94	<0.001
	80.89		41.18		35.57		35.28		44.49		40.19	
Quarrels within the family	98.53	<0.001	45.89	<0.001	44.74	<0.001	46.52	<0.001	48.88	<0.001	44.92	<0.001
	85.69		41.83		37.69		39.96		45.42		41.12	
Worse psychology	102.21	<0.001	48.00	<0.001	47.27	<0.001	47.73	<0.001	50.21	<0.001	45.57	<0.001
	89.48		42.40		39.49		42.49		46.27		42.61	
Difficulties with classes	100.21	<0.001	46.97	<0.001	45.59	<0.001	47.04	<0.001	49.27	<0.001	46.27	<0.001
	91.69		43.47		41.10		43.32		47.11		42.48	

Notes: *p*-value refers to the Mann–Whitney U tests. The first line in all the variables indicates the case of “no” while the second line refers to “yes”.

**Table 4:** Regressions results.

Variables	Model 1	Model 2
Age	−1.420 ***	−1.482 ***
	(0.260)	(0.261)
Male	6.954 ***	6.967 ***
	(0.733)	(0.732)
BMI	−0.220 **	−0.215 **
	(0.107)	(0.108)
Maternal educational level (tertiary)	1.340 **	1.359 **
	(0.552)	(0.550)
KIDMED total score	1.638 ***	1.628 ***
	(0.191)	(0.188)
Family structure (live with both parents)	3.180 ***	3.163 ***
	(1.011)	(1.014)
Night sleep duration	2.012 ***	8.107 **
	(0.400)	(3.166)
Night sleep duration/2	–	−0.426 *
		(0.218)
Screen viewing (over 4 h/day)	−1.456 **	−1.402 *
	(0.734)	(0.737)
Going out	3.120 ***	3.121 ***
	(0.431)	(0.427)
Having hobbies	0.818 ***	0.828 ***
	(0.257)	(0.257)
Constant	77.52 ***	57.04 ***
	(6.581)	(12.16)
Observations	1644	1644
R-squared	0.259	0.263

Notes: White robust standard errors in parentheses; *** *p* < 0.01, ** *p* < 0.05, * *p* < 0.1.

## Data Availability

The data presented in this study are available on request from the corresponding author.
